# A spatial inventory of freshwater macroinvertebrate occurrences in the Guineo-Congolian biodiversity hotspot

**DOI:** 10.1038/s41597-025-04471-5

**Published:** 2025-02-06

**Authors:** Emmanuel O. Akindele, Abiodun M. Adedapo, Oluwaseun T. Akinpelu, Esther D. Kowobari, Oluwatosin C. Folorunso, Ibrahim R. Fagbohun, Tolulope A. Oladeji, Olanrewaju O. Aliu, Oluwatobiloba S. Adenola, Babasola W. Adu, Francis O. Arimoro, Sylvester S. Ogbogu, Sami Domisch

**Affiliations:** 1 Leibniz Institute of Freshwater Ecology and Inland Fisheries (IGB), Department of Community and Ecosystem Ecology, Berlin, Germany; 2 Department of Zoology, Obafemi Awolowo University, Ile-Ife, Nigeria; 3 Department of Animal Biology, Federal University of Technology, Minna, Nigeria; 4 Department of Biology, Federal University of Technology, Akure, Nigeria

**Keywords:** Freshwater ecology, Limnology

## Abstract

The Guineo-Congolian region, extending from Guinea in West Africa to the central part of Africa, is considered an important biodiversity hotspot in the Afrotropics. Aside from the underreporting and underestimation of freshwater ecosystems, the challenges regarding incorrect coordinates and taxonomical inaccuracies in freshwater species occurrence data pose another major hurdle that may hinder freshwater conservation efforts in the hotspot. Hence, for any biogeographic analysis, species distribution modelling or conservation initiative, it is crucial to use datasets that are, to the largest possible extent, free of spatial and taxonomic errors. We present the final output of 8,809 occurrences consisting of 4 phyla, eight classes, 32 orders, and 1,104 species. We also added the Hydrography90m stream network attributes to the macroinvertebrate occurrence records, such that the data spans across 2,890 sub-catchments and Strahler stream orders 1–12. These records are considered valid and can be used for biogeographic analysis of freshwater macroinvertebrates in this important yet understudied freshwater biodiversity hotspot.

## Background & Summary

Freshwater biodiversity has been progressively declining over the past few decades at a higher rate than its terrestrial and marine equivalents^1–4^. The rate of freshwater species extinction over the years has led scientists to suggest that we are now living in the freshwater biodiversity crisis era^5,6^. Many freshwater species are globally threatened; for example, fish are the vertebrates with the highest rate of extinction in the 20th century^7^, and freshwater mussels are among the most threatened species globally^8^. To be more precise, the Afrotropics, Indio-Pacific, and Neotropics are experiencing the greatest declines in freshwater biodiversity worldwide^9^. Despite increasing global awareness, freshwater biodiversity in the Afrotropics still receives less conservation attention^9–11^. This is despite the fact that about 80% of the world’s freshwater macroinvertebrates are reportedly found in the tropics^12,13^, the majority of which are poorly documented^9^. This makes the need to conserve freshwater biodiversity in the region all the more urgent. Several calls to bend the curve of global freshwater biodiversity decline have been made in response to the current scenario^1,2,4,14^. The need to bend the curve cannot be overemphasised if the United Nations’ Sustainable Development Goal 15-Target 15.1 (UN SDG 15, inland biodiversity) for 2030^15^ is to be achieved. To achieve this goal and freshwater biodiversity conservation, adequate information about the many inland taxonomic groups and their geographic distributions is crucial^9,16^.

The Guineo-Congolian zoogeographical region is one of the defining features of the Afrotropical realm, which comprises the Gulf of Guinea islands as well as the tropical rainforest belt across West and Central Africa. It can be subdivided into the upper and lower sub-regions and is bordered to the north, east, and south by the African region^17^. The Guinean Freshwater Biodiversity Hotspot begins in Guinea and extends eastward to other parts of West Africa along the Gulf of Guinea, passing through the countries of Sierra Leone, Liberia, Côte d’Ivoire, Ghana, Togo, Benin, Nigeria, the southwest region of Cameroon, Sao Tome and Principe, and the Equatorial Guinea offshore islands^18^. The Congo Basin extends from the coast of the Atlantic Ocean in the west and spans through Cameroon, the Congo Republic, the Democratic Republic of Congo, Gabon, and the Central African Republic^19^. Altogether, the two forests are described as the Guineo-Congolian region^17^. The upper sub-region extends from Guinea into Benin^18^, while the lower sub-region extends from Nigeria to the central part of Africa^18,19^. The region is reported to harbour several endemic and threatened macroinvertebrates, e.g., dragonflies, molluscs, and crustaceans^20–22^.

Currently, this crucial African biodiversity hotspot lacks a comprehensive database of freshwater macroinvertebrates that covers all of the essential taxa in the countries that make up the region. Since many taxa (such as dragonflies, mayflies, stoneflies, and caddisflies) alternate between freshwater and forest ecosystems^23^, freshwater macroinvertebrates are not only an essential bioindicator of healthy freshwater ecosystems but also of healthy (riparian) forest ecosystems^24–26^. Thus, in order to achieve UN SDG 15^15^ with regard to the Guineo-Congolian region, a sufficient understanding of the geographic distribution of the region’s freshwater macroinvertebrates is essential.

To address this challenge, we compiled geographic occurrence data of freshwater macroinvertebrates from fourteen countries in the west and central parts of Africa, excluding Sao Tome and Principe, for which there was no record. We present data from published articles through personal field surveys and the Global Biodiversity Information Facility (GBIF) database from 1880 to 2024 across seven countries in the Upper Guineo-Congolian sub-region (Guinea, Sierra Leone, Liberia, Cote d’Ivoire, Ghana, Togo, and Benin) and seven countries in the Lower Guineo-Congolian sub-region (Nigeria, Cameroon, Equatorial Guinea, the Congo Republic, the Democratic Republic of the Congo, the Central African Republic, and Gabon). Given that, until now, the whole extent of the Guineo-Congolian region was considered a biodiversity hotspot, with no detailed information on how spatially distributed macroinvertebrate taxa are in the region, our new dataset provides a valuable basis to address the spatial distribution of 1,104 macroinvertebrate species, including rare and threatened species, for a more accurate delineation of freshwater biodiversity hotspots in this region. The data hence contributes to closing the knowledge gap regarding the spatial distribution of freshwater macroinvertebrates in the Guineo-Congolian biodiversity hotspot by supporting spatial freshwater biodiversity research in this region.

## Methods

We collated geographic occurrence data for freshwater macroinvertebrates in the Guineo-Congolian region from two main sources: published articles and the Global Biodiversity Information Facility (GBIF, https://www.gbif.org) platform. We collated data from published articles through search engines such as Google Scholar and PubMed. We narrowed the search to 15 countries and used the following keywords “freshwater + macroinvertebrates + <country name>”. For instance, we used ‘freshwater + macroinvertebrates + Cameroon’ to search for published findings on the occurrence records in Cameroon. In addition, we specified the following macroinvertebrate orders in the search: Ephemeroptera, Plecoptera, Trichoptera, Odonata, Unionida, and Decapoda (for instance, ‘freshwater + Ephemeroptera + Cote d’Ivoire’). We also collated data for Ephemeroptera, Plecoptera, Trichoptera, Odonata, and Unionida from the GBIF (10.15468/dl.juf33a) database^27^. Using the rgbif R-package^28,29^, we extracted data for 27 parameters which include information about taxonomic categories, geographical information, sampling time, sampling location, and source. Since Hemiptera, Coleoptera, and Gastropoda are not exclusively freshwater taxa, we used the families that are predominantly freshwater inhabitants to extract species occurrences for the taxa from the GBIF database (10.15468/dl.pbdums for Hemiptera and Coleoptera^30^, and 10.15468/dl.rsbw7w for Gastropoda^31^. The exclusively freshwater hemipteran and coleopteran families of the region include Noteridae, Hydraenidae, Hygrobiidae, Dytiscidae, Hydrophilidae, Naucoridae, Notonectidae, Gerridae, Gyrinidae, Psephenidae, Nepidae, Veliidae, Pleidae, Micronectidae, Belostomatidae, and Mesoveliidae^32–37^. Also, we collated data from the GBIF database for Platyhelminthes^38^ (10.15468/dl.k28vsg) and Decapoda^39^ (10.15468/dl.9n98gd). In addition, we used the regional freshwater biodiversity records^22^, which also contain the mollusc genera *Lymnaea*, *Ferrissia*, *Biomphalaria*, *Indoplanorbis*, *Bulinus*, *Aplexa*, and *Physa* as clues for extracting freshwater gastropod occurrences from the GBIF database. Altogether, we collected data on a variety of macroinvertebrate taxa, i.e., Annelida, Platyhelminthes, Decapoda, Odonata, Ephemeroptera, Plecoptera, Trichoptera, Hemiptera, Coleoptera, Gastropoda, and Bivalvia (Table 1). Altogether, data from published articles consist of ~11% of the occurrence records, while data from the GBIF platform consist of ~89%. Using the hydrographr R-package^40^, we extracted the corresponding sub-catchment ID, the Strahler stream order and the elevation of the Hydrography90m stream network^41^ for all occurrences (see the code in “Hydrography90m_data.r”).

**Table 1:** An overview of the freshwater macroinvertebrate database composition in the Guineo-Congolian region, consisting of 1,104 species.

Taxonomic category	Taxa
Phylum (4)	Platyhelminthes, Mollusca, Arthropoda, Annelida
Class (8)	Gastropoda, Insecta, Bivalvia, Malacostraca, Clitellata, Rhabditophora, Arachnida, Polychaeta
Order (32)	Tricladida, Hygrophila, Caenogastropoda, Odonata, Unionida, Trichoptera, Ephemeroptera, Plecoptera, Decapoda, Hemiptera, Coleoptera, Tubificida, Lepidoptera, Diptera, Araneae, Neritoidea, Rhynchobdellida, Arhynchobdellida, Cycloneritida, Littorinimorpha, Sphaeriida, Crassicletallata, Architaenioglossa, Trombidiformes, Hymenoptera, Lumbriculida, Mytilida, Venerida, Opisthopora, Trochida, Arcida, Cerithioidea
Species (1104)	Indicated in ‘GuineoCongolian_presence_subcatchments_coordinates’ file
Sub-catchments (2,890)	Indicated in ‘GuineoCongolian_presence_subcatchments_coordinates’ file

## Data Records

The file “GuineoCongolian_spdata.xls” contains a total of 8,809 occurrence records including the taxa, country code, coordinates, sampling location, sampling date, and the attributes of the Hydrography90m stream network (sub-catchment ID, elevation and Strahler stream order). We provide this file after removing duplicate occurrences, the correction of species’ spelling errors regarding their taxonomy, and after cleaning the coordinates. We also provide the original file such that potential users can apply custom data cleaning procedures if needed (“Guineo_Congolian_raw_data.xls”). All data^42^ are available at 10.18728/igb-fred-899.0.

In all, final occurrence records consist of four phyla, eight classes, 32 orders, and 1,104 species (Table 1). For the sake of data management, the macroinvertebrates are also classified into different groups (Table 2) that are often reported in the literature^20,22,43,44^. Hymenoptera and Lepidoptera, which are insect taxa, though rarely reported in the region, were also included in the major taxonomic groups. The macroinvertebrate occurrence spans across 2,890 catchments and 12 stream orders. The number of occurrence records and species richness decreased progressively from stream order 1 to 12 (Table 3). The occurrence records for the headwater streams was approximately 73% of the total records, and they also had the highest species richness compared with the medium streams and the largest streams and rivers.

**Table 2:** General Taxonomic Compositions of Freshwater Macroinvertebrates in the Guineo-Congolian Biodiversity Hotspot.

Macroinvertebrate group	Number of Species	References
Platyhelminthes	3	^38,56^
Arachnida	3	^36,49,50,57^
Coleoptera	162	^30,33,36,37,49–54,56–58,59–65^
Hemiptera	45	^30,36,37,51–54,57–63,66–75^
Diptera	17	^36,37,50,53,56–59,61,62,65,66,69,76–84^
Ephemeroptera	32	^27,37,51,58,59,62,66,69,78,79,85–88^
Odonata	499	^27,37,51,53,56,62,63,65–67,72–74,76,79,81,83,84,87,89–96^
Trichoptera	87	^27,37,49,51,53,61,63,71,73,74,77,97–100^
Plecoptera	3	^27,36,37,51,54,63,74,77,79^
Hymenoptera	1	^49^
Lepidoptera	1	^66^
Decapoda	78	^34,35,39,49–51,56,58,60,62,63,66,69–74,82,95,101–106^
Gastropoda	100	^22,31–33,36,37,50,53,62,65,69–76,80–85,87,92–95,102,104–121^
Bivalvia	48	^27,36,37,62,63,81,84,101,104,109^
Annelida	25	^36,49,50,56–62,66,69,73,76,83,115,122^

**Table 3:** Occurrence records and species richness of macroinvertebrates based on stream orders in the Guineo-Congolian region.

Order	Number of records	Species richness
1	3859	705
2	1578	496
3	992	336
4	846	356
5	437	231
6	368	238
7	261	177
8	210	162
9	195	130
10	21	21
11	22	18
12	20	12
Headwater streams (orders 1–3)	6429	865
Medium streams (orders 4–6)	1651	558
Rivers (orders 7–12)	729	370

NB: Classification system based on reference^122^.

**Fig. 1:**
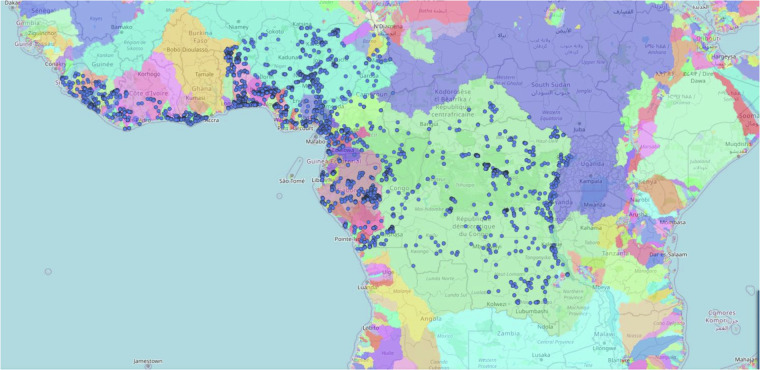
The map of freshwater macroinvertebrates in the Guineo-Congolian biodiversity hotspot, with blue points representing the 8,809 occurrence records. Coloured areas represent drainage basins of the Hydrography90m dataset. The online, interactive map can be accessed via https://glowabio.org/project/guineo-congolian_biodiversity/.

We supply the database in a single 2.37MB csv-file (“GuineoCongolian_spdata.csvs”), comprising 34 columns and 8,809 rows. The spatial map of freshwater macroinvertebrate distribution in the hotspot is shown in Fig. 1. The online, interactive map can also be visualised via https://glowabio.org/project/guineo-congolian_biodiversity/).

## Technical Validation

In some instances, especially for the published articles, the coordinates of species occurrences did not spatially match with the mentioned river, stream, or lake. In such instances, we used the GEOLocate Software (https://www.geo-locate.org/web/WebGeoref.aspx) to verify the specific location by using clues such as the name of the water body or nearby human settlement^45^. Once verified through the GEOLocate software, we supplied the coordinates for such occurrences. Also, we converted coordinates that were expressed in degrees, minutes and seconds, in published articles and unpublished field records, to decimal degree latitudes or longitudes. To ensure a taxonomic correctness of all species, we used the taxize R-package (https://github.com/ropensci/taxize). The preferred data sources for this function were Encyclopedia of Life, Zoobank registered names, and the GBIF backbone taxonomy. We further cleaned the taxized dataset using the CoordinateCleaner R-package^46^ (see also the code in “Species_data_compilation_cleaning_r”) by removing all duplicated records, and all records within 1 km (i) of country centroids, (ii) capital centroids, (iii) of zoo and herbaria, (iv) all sea records.

## Usage Notes

We provide a checklist of freshwater invertebrates in the Guineo-Congolian region with their geographical information (coordinates, countries, localities), sampling time, and the respective sub-catchment, Strahler stream order, and elevation of each record. This database could, thus, serve as a working tool for modelling the actual and potential distributions of freshwater macroinvertebrates in the region. Future predictions can also be made for the various species vis-à-vis the anthropogenic threats that characterise the Anthropocene, e.g., global climate change and deforestation^47,48^.

**Fig. 2:**
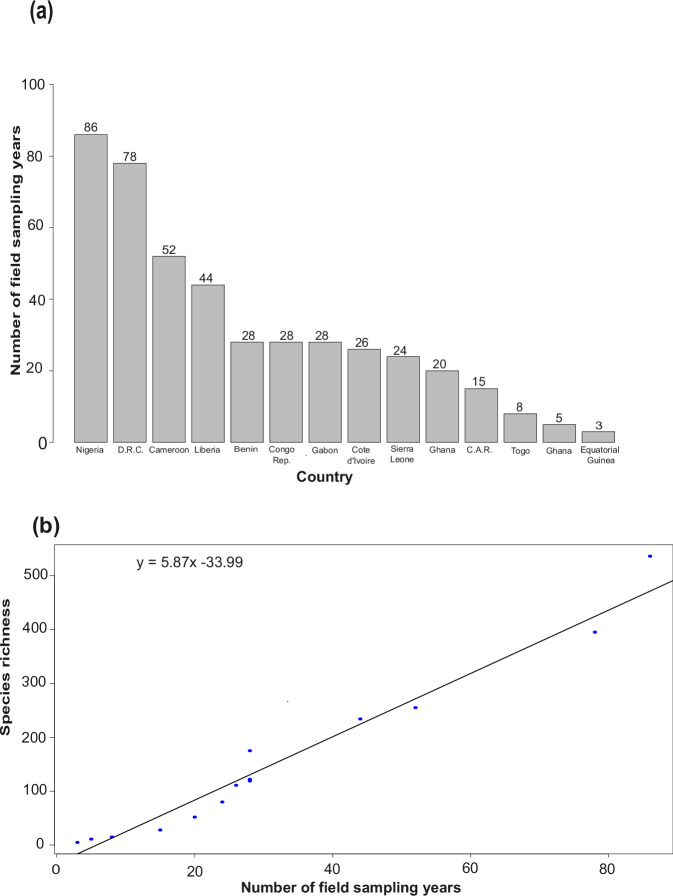
(**a**) Number of sampling years in each country in the region. (**b**) A generalized linear model of species richness vs number of sampling years.

We highlight that the spatial patterns shown in the distribution of freshwater macroinvertebrates in the Guineo-Congolian region indicate that some countries are underreported, especially countries in the upper Guineo-Congolian sub-region. The trend reported in this study may not necessarily be an indication of biodiversity richness in each country or sub-region, but rather of the scientific efforts deployed in field samplings and recordings over the years (Fig. 2a). A generalised linear model of the number of field sampling years and species richness indicates a very strong and direct relationship in the region: the higher the sampling effort, the higher the species richness (Fig. 2b). This is a clear indication that freshwater invertebrates in such countries (e.g., Guinea, Sierra Leone, Togo, Benin, Equatorial Guinea, and Central African Republic) need to be intensively sampled. Recent field samplings in some previously unreported Guineo-Congolian freshwater systems led to the discovery of some threatened macroinvertebrates. Such species include *Pentaphlebia stahli*^16^, *Africocypha centripunctata*, and *Allocnemis vicki*^49^, which could have gone unreported if they were not sampled.

The importance of headwater streams as biodiversity refuges is also established by the species richness of freshwater macroinvertebrates among the stream orders. Threatened species such as *P. stahli*, *A. centripunctata*, and *A. vicki* were recorded in the headwater streams (Strahler stream orders 1–3). This buttresses the assertions of various scientists that the contributions of headwater streams to the overall biodiversity of river networks are quite enormous in terms of species richness and as habitats for rare and threatened species^50,51^. The disproportionately low numbers of macroinvertebrate records and species in large rivers of the hotspot could also be due to logistic reasons, since smaller streams are wadable and are thus easier to sample than large rivers. However, it would be important to highlight that large rivers may harbour important undescribed freshwater species in the Guineo-Congolian region.

There is a clear indication that if the sampling effort in these countries and sub-regions is intensified, especially for under-reported but critical macroinvertebrate taxa like Ephemeroptera-Plecoptera-Trichoptera (EPT), Unionida, and Decapoda, more freshwater macroinvertebrate species could be detected or described (see Fig. 2b). The EPT taxa in particular are commonly used as bioindicators for freshwater ecosystem health in numerous national monitoring programs^11,52^. The bulk of the data on freshwater macroinvertebrates in the region is on Odonata, which has been intensively studied by odonatologists and citizen scientists since they are ubiquitous in both freshwater (as larvae) and riparian (as adults) systems, unlike other aquatic insect taxa^21,53,54^. It is also worth noting that many occurrence records (EPT and other taxa) in published articles for the region were only identified at the family or generic level. Such records were excluded from our database since the focus was on reporting the species. Hence, this important biodiversity hotspot can still be considered data-deficient in the aforementioned under-reported taxa.

Since it is hypothesised that sample size and frequency are positively correlated with species richness^55^, the importance of intensive macroinvertebrate sampling in under-reported Guineo-Congolian countries, and on the under-reported taxa cannot be overemphasised.

## Supplementary information

Guineo-Congolian macroinvertebrate data

Guineo-Congoliam macroinvertebrate raw data

Code for species data

Code for Hydrography 90m data and bioclimatic variables

## Data Availability

The data^42^ (GuineoCongolian_spdata.csv) as well as the codes (“Species_data_compilation_cleaning.r” and “Hydrography90m_data.r”) are officially deposited at 10.18728/igb-fred-899.0.
